# Atypical *URA5* gene restriction fragment length polymorphism banding profile in *Cryptococcus neoformans* strains

**DOI:** 10.1007/s12223-019-00699-y

**Published:** 2019-04-08

**Authors:** Magdalena Florek, Jarosław Król, Anna Woźniak-Biel

**Affiliations:** 1 Department of Pathology, The Faculty of Veterinary Medicine, Wrocław University of Environmental and Life Sciences, Norwida 31, 50-375 Wrocław, Poland; 2 Department of Epizootiology and Clinic of Birds and Exotic Animals, The Faculty of Veterinary Medicine, Wrocław University of Environmental and Life Sciences, pl. Grunwaldzki 45, 50-366 Wrocław, Poland

## Abstract

*URA5*-RFLP is one of the most widely used genotyping methods relating to *Cryptococcus neoformans* and *C. gattii* consensus genotype nomenclature. In order to identify a molecular type, this method uses a visual comparison of digested PCR products of tested and reference strains, therefore any anomaly in RFLP patterns of studied isolates makes recognition difficult or impossible. This report describes a strain of VNIV type showing an atypical *URA5-*RFLP pattern as well as a group of AD hybrids displaying the same anomaly. The atypical RFLP pattern is the result of a point mutation and emergence of a new restriction site. Emergence of the allele presenting a new banding pattern may lead to misidentification using the *URA*5-RFLP technique; the results of this study as well as the literature data may suggest the spread of the allele in the environment.

## Introduction

*Cryptococcus neoformans/C. gattii* species complex is associated with life-threatening infections in humans and animals worldwide. These pathogens have been isolated from different environmental sources, mainly trees, soil and pigeon droppings (Mitchell et al. 2011) that may serve as a source of infection. For the last several decades, genetic heterogeneity within the complex has been demonstrated using a wide range of molecular typing methods (Meyer et al. 2009). In 2007, the ISHAM Working Group for Genotyping of *C. neoformans* and *C. gattii* recognised that the various molecular typing methods reveal corresponding major genotypes for these fungi (Meyer et al. 2009). A consensus has been established for the genotype nomenclature of *C. neoformans* and *C. gattii* which divides *C. neoformans*/*C. gattii* species complex into eight major molecular types, namely VNI and VNII (representing *C. neoformans* var. *grubii*, serotype A), VNIV (*C. neoformans* var. *neoformans*, serotype D) and VNIII (the hybrid of these two species, serotype AD) as well as VGI, VGII, VGIII and VGIV (C. *gattii,* serotype B or C) (Meyer et al. 2009). In 2015, Hagen et al. proposed these molecular types as separate species (Hagen et al. 2015), but since there is not a complete consensus among the scientific community regarding this subject, the former nomenclature was used in this paper. Restriction fragment length polymorphism analysis of the orotidine monophosphate pyrophosphorylase gene (*URA5*-RFLP) is one of the typing techniques used in molecular epidemiological studies of fungi belonging to the complex*.* In this method, the molecular type is assigned visually, by comparing electrophoretograms of digested PCR products of tested isolates and standard strains representing the major molecular types (Meyer et al. 2003). Therefore, emergence of strains showing atypical digestion patterns may make it difficult or even impossible to correctly recognise them using *URA5*-RFLP analysis.

**Fig. 1:**
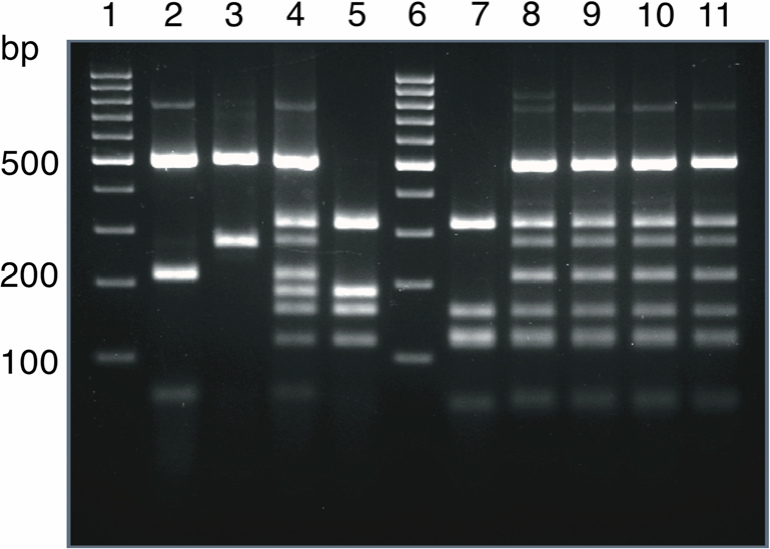
*URA5*-RLFP profiles of reference and atypical strains. Lanes: 1 and 6—molecular marker (Gene Ruler 100 bp Plus DNA Ladder, Thermo Scientific Waltham, MA, USA); 2–5 reference strain profiles (2—VNI, 3—VNII, 4—VNIII, 5—VNIV); 7–11 atypical strains (7—atypical VNIV, 8–11—atypical VNIII)

As it was reported in Meyer et al. (2003), the restriction profile of the reference VNIV strain (Fig. 1, lane 5) displays a typical four-band pattern. According to restriction map analysis of the *URA5* gene sequence of the reference VNIV strain CBS 10079 (WM 629), the banding pattern consists of DNA fragments of about 115, 156, 186, and 318 bp in size.

The present report describes a group of isolates showing an atypical *URA5*-RFLP banding profile. The strains were isolated during an environmental survey conducted in the territory of Lower Silesia, Poland, between July 2014 and January 2018.

## Materials and methods

Environmental samples were collected in the form of swabs taken from tree holes and pigeon droppings, or obtained directly from pigeons. Specimens obtained directly from birds (throat, crop and cloaca swabs) were collected during the annual, routine clinical examinations of health status in pigeon flocks. Both the tree and excreta samples were placed in tubes containing 3 mL of sterile saline solution, vortexed for 2 min and left for 10 min in order to let the suspension settle. Then the supernatants were diluted 1:10 with saline. Two sets of plates containing Niger seed agar (NSA) were inoculated with 100 μL of the supernatant and its dilution, respectively. The swabs taken from birds were inoculated directly onto the NSA. Plates were incubated at 30 °C for up to 14 days, then colonies showing different degrees of melanisation were subcultured in order to purify the cultures. The isolates were tested using India Ink staining and prepared for further studies. DNA extraction was performed using the MasterPure™ Yeast DNA Purification Kit (Epicentre Biotechnologies, Madison, WI, USA), according to the manufacturer’s instructions. The DNA of tested strains was analysed using multilocus sequence typing (MLST) and/or *URA5*-RFLP methods. MLST analysis was performed according to the ISHAM consensus scheme concerning seven genetic loci (*CAP59*, *GPD1*, *LAC1*, *PLB1*, *SOD1*, *URA5*, and *IGS*1) (Meyer et al. 2009). For amplification of the *SOD1* gene of *C. neoformans* var. *neoformans* strains, an alternative reverse primer was used, as described in Sanchini et al. (2014); PCR conditions for *LAC*1 locus, presented by Litvintseva et al. (2006), were applied instead of those given in the consensus. PCR products were purified and sequenced (DYEnamic ET terminator cycle sequencing kit ABI Prism™, Amersham Biosciences Europe GmbH, Germany). Forward and reverse sequences were assembled using BioEdit v7.2.0 (http://www.mbio.ncsu.edu/BioEdit/bioedit.html) and then analysed by BioloMICS Polyphasic Identification Software (http://mlst.mycologylab.org/cneoformans); as a result, allele types (AT) of each gene as well as sequence types (ST) and molecular types (MT) of the tested strains were assigned. The *URA5*-RFLP technique was conducted according to Meyer et al. (2003) PCR products were double digested with Cfr13I (Sau96I) and HhaI enzymes (Thermo Fisher Scientific, Waltham, MA, USA) for 16 h and separated in 3% agarose gel at 100 V for 3 h. RFLP patterns of the tested strains were determined visually by comparison with standard strains (CBS 8710-VNI, CBS 10084-VNII, CBS 132-VNIII and CBS 10079-VNIV). Restriction maps of the tested and reference strains were prepared using BioEdit v7.2.0 software. As a reference, a sequence of the *URA*5 gene of the WM 629 (CBS10079) strain was used. Since a sequence matching the exact length of the PCR product obtained in the *URA*5-RFLP method was not available in GenBank, a shorter sequence was chosen (GenBank accession number KC568724). Sero- and mating-types of all tested isolates were established using a PCR-based method of amplification of serotype-specific and mating-type-specific *STE*20 gene, described by Li et al. (2012). The following strains were used as positive controls: CBS 10084 (Aα), CBS 132 (AαDa), IUM 96–2828 (Aa) and CBS 10079 (Dα). Additionally, primers specific for *LAC*1 locus of A or D types, designed by the same authors, were used to enable discrimination of a serotype (Li et al. 2012).

## Results

**Fig. 2:**
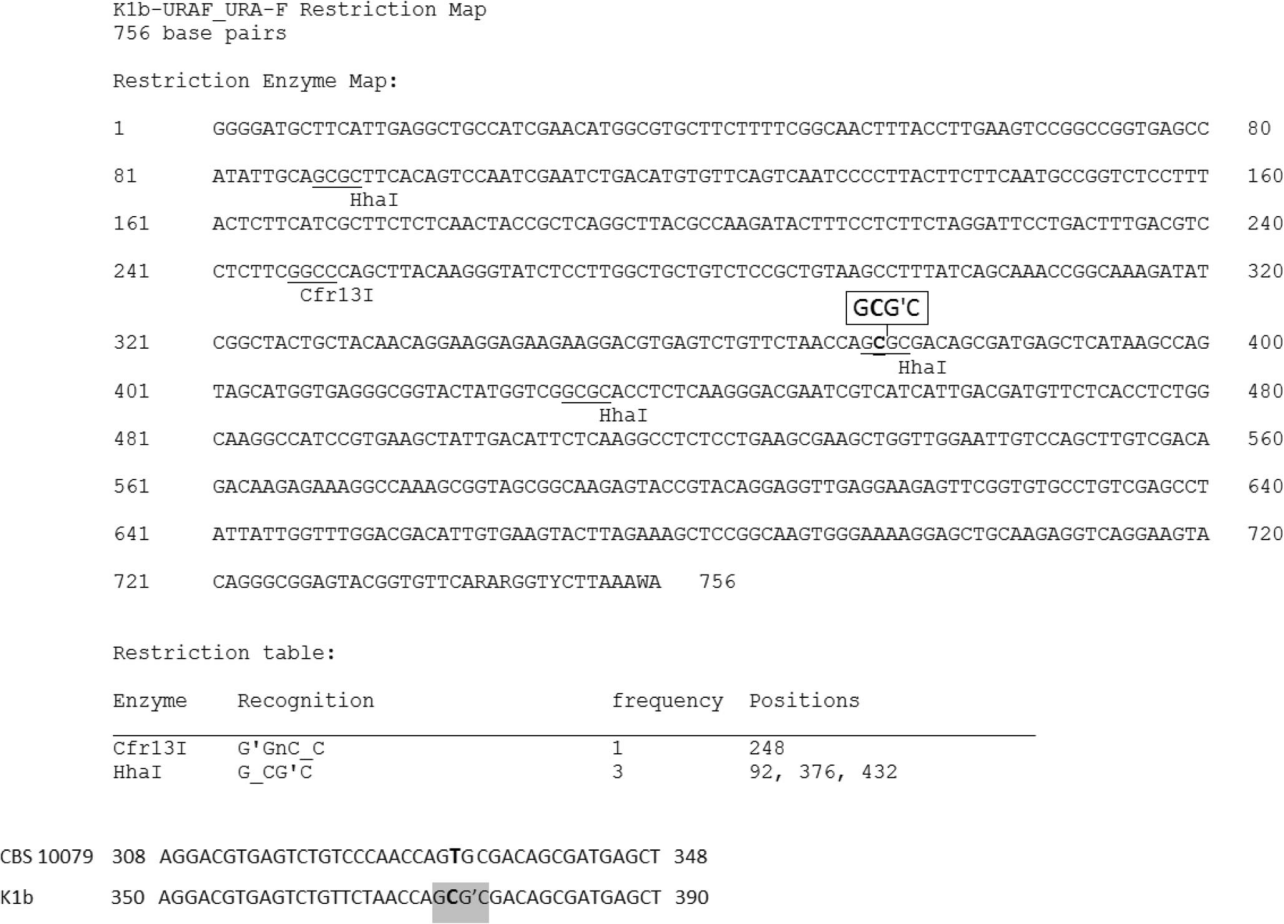
Restriction map of K1b strain *URA*5 gene prepared using BioEdit v7.2.0 (top); comparison of CBS 10079 and K1b strains’ sequence fragments. In K1b sequence point mutation (bold; position 374) as well as recognition sequences in the new restriction site are indicated (bottom)

The *URA5*-RFLP analysis revealed that one (K1b) of the strains isolated during the survey displayed an atypical banding profile. According to MLST analysis, the strain was recognised as VNIV-ST514, but instead of a four-band pattern, typical of the VNIV type, it displayed five bands (56, 117, 128, 156 and 348 bp). The anomaly emerged due to the presence of an additional restriction site for the HhaI enzyme (5′…G**C**G↓C…3′; Fig. 2) at position 376, resulting from the substitution of thymine for cytosine at position 374 (corresponding to position 332 in used CBS 10079 sequence). As a result, the digested PCR product of the atypical strain (Fig. 1, lane 7) lost the 184-bp band, which was cut and replaced by two smaller fragments (56 and 128 bp). The mutation-bearing allele of the *URA5* gene represented AT #32, according to the MLST analysis. Interestingly, among other strains isolated in the territory of Lower Silesia, four isolates of VNIII hybrids were identified, all presenting a *URA5-*RFLP pattern anomaly similar to the one described in the atypical VNIV isolate (Fig. 1, lanes 8–11). The sero- and mating-type assays, assigned the strain K1b as a Dα type, while all the hybrids were recognised as AαDα. Both *LAC*1A and *LAC*1D PCR products were obtained with respect to all the hybrids. Strains described in this study were deposited in the Polish Collection of Microorganisms (https://www.iitd.pan.wroc.pl/en/PCM/index.html; accession numbers: PCM 2996, PCM 2997, PCM 2998, PCM 2999, PCM 3000) and additionally studied K1b strain sequences were sent to the Cryptococcus MLST database (www.mycologylab.org).

The K1b strain was isolated in October 2017 from an oak tree situated in a forest, about 16 km southwest of Wroclaw, while the hybrid strains were obtained between December 2015 and February 2016 from domestic pigeons living in locations about 97 and 14 km distant from the collection site of the strain K1b.

## Discussion

This report is the first to identify a group of VNIV and hybrid strains of *C. neoformans* species complex that due to a point mutation may cause difficulties in interpretation of *URA5*-RFLP assay results. A similar situation was already reported in the case of a group of *C. gattii* strains of type VGIII, which due to a point mutation, gave the same RFLP pattern as that observed normally in type VGIV (Trilles et al. 2014). Strains sharing the same *URA*5 AT#32 as described here were also isolated in Belgium, Germany, Italy and Turkey (Cogliati et al. 2016), but the molecular type of those strains was established using the multiplex PCR instead of *URA*5-RLFP technique, and the restriction anomaly presented in our study might have gone unnoticed. The presence of the *URA*5 allele type in Western and Southern Europe (Cogliati et al. 2016), along with the fact that in our study, the allele was isolated in Poland, collected over the course of 3 years and from different locations, may suggest its wider spread in the environment. This allele was identified in 8.7% of VNIV strains tested during the survey performed by us and in 6 of the 83 (7.2%) isolates of this molecular type analysed by Cogliati et al. (Cogliati et al. 2016). *C. neoformans* var. *neoformans* has not been studied as extensively as *C. neoformans* var. *grubii*, yet recent studies suggest that it is characterised by a variability higher than that observed in its sibling variant (Cogliati et al. 2016; Desnos-Ollivier et al. 2015). Further studies concerning this population are required, especially in Europe where the frequency of its isolation seems to be the highest (Cogliati 2013), in order to establish its genetic structure and epidemiology, as well as to find whether or not the observed variability may pose a problem or limitation with respect to widely used genotyping methods.
